# *In-Silico* Modeling of the Mitotic Spindle Assembly Checkpoint

**DOI:** 10.1371/journal.pone.0001555

**Published:** 2008-02-06

**Authors:** Bashar Ibrahim, Stephan Diekmann, Eberhard Schmitt, Peter Dittrich

**Affiliations:** 1 Bio System Analysis Group, Institute of Computer Science, Friedrich-Schiller-University Jena, Jena, Germany; 2 Jena Centre for Bioinformatics (JCB), Jena, Germany; 3 Leibniz Institute for Age Research (FLI)-Fritz Lipmann Institute, Jena, Germany; University of Edinburgh, United Kingdom

## Abstract

**Background:**

The Mitotic Spindle Assembly Checkpoint (^M^SAC) is an evolutionary conserved mechanism that ensures the correct segregation of chromosomes by restraining cell cycle progression from entering anaphase until all chromosomes have made proper bipolar attachments to the mitotic spindle. Its malfunction can lead to cancer.

**Principle Findings:**

We have constructed and validated for the human ^M^SAC mechanism an *in silico* dynamical model, integrating 11 proteins and complexes. The model incorporates the perspectives of three central control pathways, namely Mad1/Mad2 induced Cdc20 sequestering based on the Template Model, MCC formation, and APC inhibition. Originating from the biochemical reactions for the underlying molecular processes, non-linear ordinary differential equations for the concentrations of 11 proteins and complexes of the ^M^SAC are derived. Most of the kinetic constants are taken from literature, the remaining four unknown parameters are derived by an evolutionary optimization procedure for an objective function describing the dynamics of the APC:Cdc20 complex. MCC:APC dissociation is described by two alternatives, namely the “Dissociation” and the “Convey” model variants. The attachment of the kinetochore to microtubuli is simulated by a switching parameter silencing those reactions which are stopped by the attachment. For both, the Dissociation and the Convey variants, we compare two different scenarios concerning the microtubule attachment dependent control of the dissociation reaction. Our model is validated by simulation of ten perturbation experiments.

**Conclusion:**

Only in the controlled case, our models show ^M^SAC behaviour at meta- to anaphase transition in agreement with experimental observations. Our simulations revealed that for ^M^SAC activation, Cdc20 is not fully sequestered; instead APC is inhibited by MCC binding.

## Introduction

The growth of all organisms requires that the genome is accurately replicated and equally partitioned between two cellular progenies. In eukaryotes, the duplication of chromosomes, the separation of sister chromatids, and their segregation to opposite poles of the cell prior to cytokinesis are features of the cell cycle and grant maintenance of genomic integrity [1]. Eukaryotic cells have evolved a surveillance mechanism for DNA segregation, the ^M^SAC. This checkpoint blocks anaphase onset and prevents exit from mitosis until all chromosomes are properly attached and have aligned on the mitotic spindle. Its malfunction leads to cell death [2]–[4], generates aneuploidy [5]–[7] (deviation from euploidy is seen in 70–80% of all types of human cancers [8]), might facilitate tumorgenesis [9], [10] and aging [11], and might contribute to cancer [12]–[14] (reviewed in [9], [15]–[19]).

### Current models of the ^M^SAC

Despite considerable experimental knowledge, the ^M^SAC has not yet been modeled at a detailed molecular level. Doncic et al. [20] compared several mechanisms that could account for the inhibition of the APC:Cdc20 complex in yeast. They noticed that the design of the ^M^SAC network is limited by physical constraints imposed by realistic diffusion constants and the relevant spatial and temporal dimensions in the yeast cell. Designing a simplified model of radial symmetry, they observed that amplifying the signal through the release of a diffusible inhibitory complex can describe checkpoint function. Nevertheless, their model does not fully take into account the molecular complexity. A similar approach was presented by Sear et al. [21]. They investigated two mechanisms for ^M^SAC in metazoan cells: one involves free diffusion and sequestration of cell cycle regulators requiring a two-stage signal amplification cascade. The second mechanism involves spatial gradients of a short-lived inhibitory signal that propagates by diffusion and primarily by active transport along spindle microtubules. Both mechanisms might act in parallel. Mathematical modeling of cell cycle control in budding yeast was analyzed in more details in [22], however not focusing on ^M^SAC. A model for the exit from mitosis [23] describes the control of the checkpoint, however not considering BubR1 (Mad3 in yeast) nor MCC.

Here, we suggest a kinetic model based on a set of time dependent nonlinear ordinary differential equations for protein concentrations. The model describes the ^M^SAC on the molecular level. It focuses on ^M^SAC control in mitosis at metaphase to anaphase transition; it does not include exit from mitosis (e.g., Cdh1). The Mad1/Mad2 action and Cdc20 inhibition is described by a recently developed mathematical model [24] based on the biochemical Template Model [25], [26]. The description of MCC formation and APC inhibition is based on results from biochemical experiments [15], [27]–[31]. We present the chemical basis of the reactions and explain the chemical reaction equations in detail. Then, we describe the corresponding ordinary differential equations and their mathematical treatment.

It is still unclear how the MCC:APC complex falls apart and how the APC:Cdc20 complex is formed afterwards. Therefore we consider here two alternative pathways in our ^M^SAC Model, the “Dissociation” and the “Convey” variants, differing in one reaction: either the MCC:APC complex dissociates into the MCC and the APC (“Dissociation variant”), or, alternatively, Cdc20 being a member of the MCC remains at the APC and only the other MCC complex members leave the MCC:APC (“Convey variant”). We noticed that checkpoint behavior requires that the dissociation of the MCC:APC is regulated by microtubule attachment. For this purpose we introduced a factor for the attachment dependent control of the associated reactions. We compared the controlled versus the uncontrolled case. Those resulting model variants that describe checkpoint function properly are validated by comparison to ten different deletion and over-expression experiments taken from literature. From our model calculations we conclude that the meta- to anaphase transition and the APC are not inhibited by Cdc20 sequestering but instead the APC is bound and blocked by the MCC.

## Methods

### Biochemical background

Our model incorporates three ^M^SAC-related mechanisms: the Template Model, the (kinetochore dependent) MCC formation, and the APC inhibition. Their biochemical details will be explained in the following.

### Mad2 Template Model

DeAntoni et al. [25] proposed the “Template Model” explaining the mechanism of Mad2 recruitment to the kinetochore during checkpoint activation and subsequent transfer to sequester Cdc20. Recent work by Vink et al. [27] and Mapelli et al. [26] provide additional support for the Template Model. Moreover, this model has been confirmed by Nezi et al. [32], and is entirely consistent with recent Fluorescence Recovery After Photobleaching (FRAP) data [27], [28]. The Template Model [25] is superior and more solid than the Exchange Model [33], which we confirmed in a recent *in silico* study [24] (for comparison and details see [25]–[27], [32], [34], for reviews see [35]–[39]).

The Mad2 Template Model is described by the reaction equations Eqs. (1)–(3) (see chemical reaction scheme, below). It is assumed that Mad1 and C-Mad2 form a stable core complex Mad1:C-Mad2 at unattached kinetochores [25]. In our nonlinear ordinary differential equations (ODEs) model, we assume that this process has already been completed. Therefore, there is no free Mad1. Equation (1) describes how the Mad1:C-Mad2 core complex binds additional molecules of O-Mad2 through formation of conformational heterodimers between the C- Mad2 subunit of the Mad1:C-Mad2 complex and O-Mad2. Upon Mad1:C-Mad2 binding, O-Mad2 adopts an intermediate conformation (O-Mad2*), which can quickly and efficiently bind Cdc20 and switch to the C-conformation. This process is documented by Eq. (2): Cdc20 binding to the complex Mad1:C-Mad2:O-Mad2* leads to the conversion of O-Mad2* to C-Mad2 forming together with Cdc20 the complex Cdc20:C-Mad2; Cdc20:C-Mad2 is assumed then to dissociate off Mad1:C-Mad2 [40]. Finally, we assume that the Cdc20:C-Mad2 complex can dissociate into Cdc20 and O-Mad2 (Eq. (3)).

### MCC formation

Equations (4) and (5) describe the formation of the MCC, which contains Mad2, Bub3, BubR1 and Cdc20 in apparently equal stoichiometries [41]–[44]. Bub3 associates quite stably with BubR1 [18], [41], [45]. This interaction is constitutive and is required for the localization of BubR1 to the kinetochores during mitosis. Like for the Mad1:C-Mad2 complex, we do not model the dynamics of the formation of the BubR1:Bub3 complex. BubR1 cannot bind Mad2 directly [40]. Moreover, BubR1 does not form a ternary complex with Mad2 and Cdc20. Two Cdc20 binding sites were identified on BubR1 [46], [47]. Binding of the N-terminal region of BubR1 to Cdc20 requires prior binding of Mad2 to Cdc20 [47]. Consistently, the Bub3:BubR1 complex can bind to Cdc20:C-Mad2 in order to form the MCC (Eq. (4), rate constants k_4_ and k_-4_). The other site of BubR1 (between residues 490 and 560) can bind Cdc20 tightly regardless of Mad2 being bound to Cdc20 [47]. Thus, BubR1 can form a ternary complex with Bub3 and Cdc20 (Eq. (5)) which however has no inhibitory activity at the APC (unpublished data [41]). Equation (6) and its low rate were mentioned by Musacchio & Salmon [15].

### APC inhibition

The MCC is considered to be essential for ^M^SAC function, because it binds and inhibits the APC [41]–[43], [48]–[55]. However, MCC inhibits only the mitotic, and not the interphase APC [56]. The interaction between APC and MCC is quite labile in the absence of unattached kinetochores [41]. How the MCC inhibits APC activity is poorly understood [15]. The MCC might bind to the APC as a pseudosubstrate due to a KEN-box motif in BubR1 [40], [53], [57], [58]. This indicates that the MCC needs to disassemble from the APC at metaphase to elicit anaphase [40], [53]. Bub1 and Aurora-B kinase contribute directly to the formation of a complex of the MCC with the APC [53] (represented by k_7_ in Eq. (7)). Unattached kinetochores might sensitize the APC for inhibition by the MCC [20], [21], [38], [41] (represented by u in Eq. (7)). In addition to kinetochore attachment, tension is important for ^M^SAC inactivation [59], [60]: if both sister kinetochores attach to microtubules from the same pole, not enough tension is generated and microtubules kinetochore attachment is destabilized to correct the problem [15]. This destabilization depends on Aurora-B kinase [56], [61]–[63]. Again, these effects are subsumed by the switching parameter u. For complex dissociation we consider two model variants:

In the “Dissociation variant”, we assume that MCC binds to APC and that this binding is reversible (Eq. (7^a^)). Free Cdc20 has to bind reversibly to APC (Eq. (8)), effectively competing with MCC.

In the “Convey variant”, we do not assume that the APC:MCC complex simply dissociates into APC and MCC, but that the MCC complex falls apart so that the Cdc20 contained in the MCC complex can bind to the APC (Eq. (7^b^)).

### Control by attachment

Several reactions in the reaction scheme are controlled by the attachment of microtubules to the kinetochore which is realized by the factor u present in several reaction equations [64]. Factor u represents the function of proteins like p31^comet^, UbcH10, and Dynein (and its activator Spindly [65]). p31^comet^ prevents further Mad2 turnover on Mad1 and neutralizes the inhibitory activity of Cdc20-bound Mad2 [26], [66]–[68]. Catalytically active UbcH10 can promote the release of checkpoint proteins from APC [69]. Dynein [70] removes the Mad1:C-Mad2 2:2 complex from the kinetochore site after microtubule attachment. Thus, p31^comet^, UbcH10, and Dynein work in concert during checkpoint inactivation.

Also the MCC:APC complex dissociation might be attachment controlled. We therefore introduced the factor u′ in Eq. (7^a^) and Eq. (7^b^), allowing us to compare the uncontrolled (u′ =  1) with the controlled (u′ = 1−u, i.e., u′ = 0 before and u′ = 1 after attachment) case. The switching parameter u′ might represent the protein function of Usp44, which deubiquitinates the APC co-activator Cdc20 both *in vitro* and *in vivo*, and thereby directly counteracts the APC-driven disassembly of Mad2:Cdc20 complexes [71], [72].

### Chemical reaction scheme

In our model of the ^M^SAC mechanism, 9 biochemical reaction equations describe the dynamics of the following 11 species: Mad1:C-Mad2, O-Mad2, Mad1:C-Mad2:O-Mad2*, Cdc20, Cdc20:C-Mad2, Bub3:BubR1, MCC, Bub3:BubR1:Cdc20, APC,MCC:APC, and APC:Cdc20. Because the dissociation of the MCC:APC complex is not known in detail, we introduce two variants for the reaction equation for MCC:APC dissociation.

**Figure 1:**
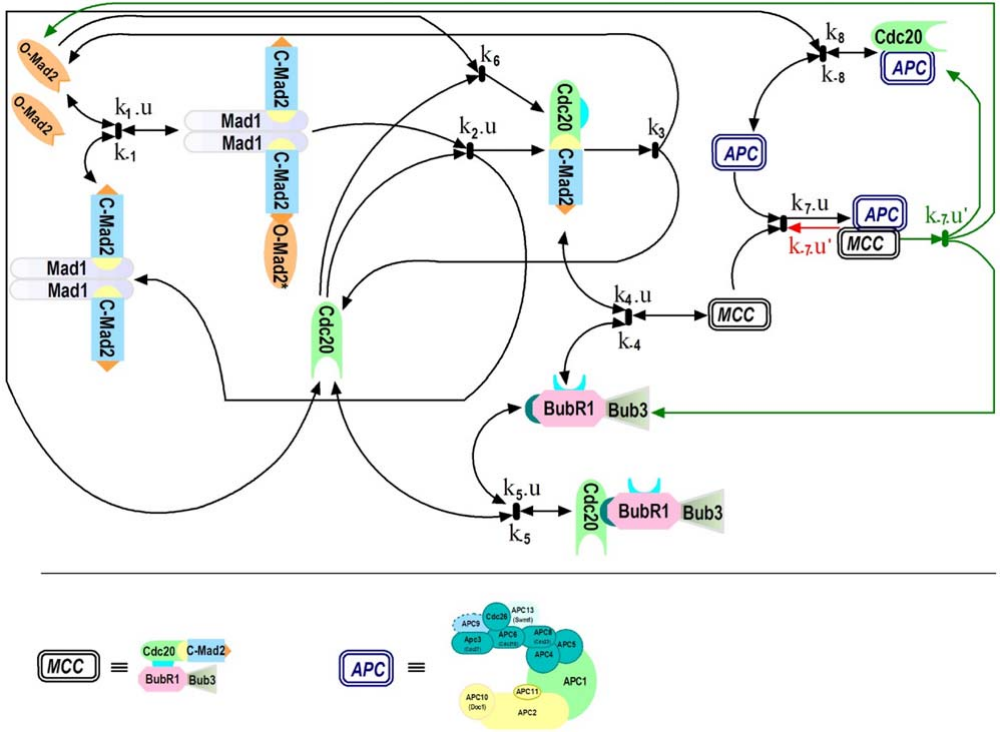
Schematic network of the ^M^SAC model. The arrows describe the interactions between the proteins and complexes. Red lines represent the Dissociation variant, green lines represents the Convey variant, while the black arrows are common to both. The switching parameter u models the effect of the attachment. We set u = 1 for the unattached case and u =  0 for the attached case. We set u′ = 1 for the uncontrolled scenario and u′ = 1−u for the controlled scenario (Table 1). The model incorporates three central control mechanisms, namely Mad1/Mad2 induced Cdc20 sequestering based on the Template Model, MCC formation, and APC inhibition. These sub-systems can be red from left to right. Nine biochemical reaction equations describe the interactions of 11 species: Mad1:C-Mad2, O-Mad2, Mad1:C-Mad2:O-Mad2*, Cdc20, Cdc20:C-Mad2, Bub3:BubR1, MCC, Bub3:BubR1:Cdc20, APC, MCC:APC, and APC:Cdc20. Below the network, the subunits of MCC as well as APC are depicted.

The Dissociation variant is defined by the following reaction rules (Figure 1, red lines):
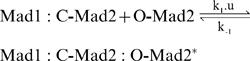
(1)

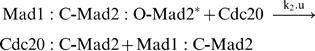
(2)

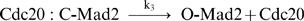
(3)

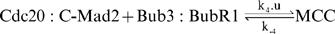
(4)

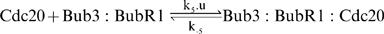
(5)

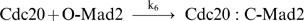
(6)

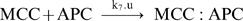
(7)

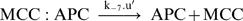
(7a)

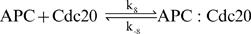
(8)


**Table 1:** Model Variants

Scenario	Model variants	Reaction rules	Control of MCC:APC Dissociation
Uncontrolled	Dissociation	Eqs. (1)–(7), (7^a^), (8)	u′ = 1
Controlled	Dissociation	Eqs. (1)–(7), (7^a^), (8)	u′ = 1−u
Uncontrolled	Convey	Eqs. (1)–(7), (7^b^), (8)	u′ = 1
Controlled	Convey	Eqs. (1)–(7), (7^b^), (8)	u′ = 1 − u

The reaction rules defining the second variant, the Convey variant, are different from this set by replacing the back reaction Eq. (7^a^) by Eq. (7^b^) (Figure 1, green lines):
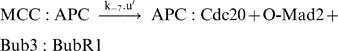
(7b)


Both variants are controlled by the switching parameters u and u′. They represent a signal generated by the unattached and attached kinetochores, respectively. If the kinetochore is unattached, we set u = 1, otherwise u = 0. For instance, formation of Mad1:C-Mad2:O-Mad2* (Eq. (2)) can only take place as long as the kinetochores are unattached [25].

The switching parameter u′ represents an additional hypothetical control, whose biochemical realization is described above. For each of the two dissociation variants, we therefore considered two scenarios: In the first, we assume that this control does not exist by setting u′ = 1. In the second, we assume that there is a control by setting u′ = 1−u. This is summarized in Table 1:

### Mathematical treatment and simulation

By applying general principles of mass-action kinetics, we converted the reaction rules into sets of time dependent nonlinear ordinary differential equations (ODEs) for the Dissociation variant (Text S1, Eqs. (D.1)–(D.11)) and for the Convey variant (Text S1, Eqs. (C.1)–(C.11)).

For the rate constants k_i_, we selected experimentally determined values, if available (Table 2). In the other cases, we selected representative values exemplifying their whole physiologically possible range. We also fitted unspecified parameters by minimizing an APC:Cdc20 concentration dependent objective functional (Text S1, C), taking into account the range of parameter values from experiments [15], [27], [40], [55], [73].

**Table 2:** Model parameters

Parameters	Comments and References
**Species initial concentration**	
[Cdc20] = 2.2* 10^−7^M	[40], [55], [71]
[Mad2]_total_ = 2* 10^−7^M	[40], [55], [71]
[BubR1:Bub3] = 1.3* 10^−7^M	[40], [55]
[APC] = 0.9*10^−7^M	[69]
Other species are zero	
**Species concentration ratios**	
25% of [Mad2]_total_ associated with Mad1, [Mad1:C-Mad2] = 25%[Mad2]_total_	[33], [40]
[O-Mad2] = 75%[Mad2]_total_	[40], [71]
**Model–Parameters**	
k_1 = _2*10^5^ M^−1^s^−1^	[31]
k_-1 = _2*10^−1^ s^−1^	[31]
K_2 = _10^8 ^M^−1^s^−1^	[24]
K_3 = _10^−2 ^s^−1^	[24]
K_4 = _10^7 ^M^−1^s^−1^	[62]
k_-4 = _10^−2^ s^−1^	[62]
K_5 = _10^4 ^M^−1^s^−1^	[62]
k_-5 = _10^−1 ^s^−1^	[62]
K_6 = _10^3 ^M^−1^s^−1^	[15]
K_7 = _10^8 ^M^−1^s^−1^	This study
k_-7 = _8*10^−2^ s^−1^	This study
K_8 = _5*10^6^ M^−1^s^−1^	This study
k_-8 = _8*10^−2^ s^−1^	This study

In a typical simulation, we initialized all reaction partners according to Table 2 and numerically integrated the ODEs until steady state was reached using u = 1 (denoting unattached kinetochores). Then we set u = 0 (kinetochores attached) and continued integrating the ODEs until we again reached a steady state.

The minimum concentration of APC:Cdc20 before attachment and the speed of recovery after attachment (recovery time) are criteria for ^M^SAC function and were analyzed to compare the models. Deduced from the biochemical data (see above), the APC:Cdc20 concentration must be low before and the recovery must be fast after attachment.

## Results

We developed a theoretical model of the human biochemical mitotic checkpoint at meta- to anaphase transition. As described in the literature, many proteins contribute to checkpoint function. The key players and their interactions are captured by the reaction equations introduced in the previous section. We transformed these equations into ODEs and selected specific values for the initial concentrations and rate constants from the literature and our previous publications (summarized by Table 2). For only four values we could not identify specific data in the literature. We obtained these values by optimizing the properties of the model according to the APC:Cdc20 level: this complex level should be low in metaphase and high in anaphase; furthermore, the switching should be fast (see Text S1 for details). We found good behavior of the model network for the values k_7_ = 10^8^M^−1^s^−1^, k_−7_ = 8*10^−2^s^−1^, k_8_ = 5*10^6^M^−1^s^−1^, and k_−8_ = 8*10^−2^s^−1^.

### ^M^SAC Model behavior

We analyzed the dynamics of the model integrating 11 proteins and complexes of the ^M^SAC. The literature does not provide a clear view, yet, about how the MCC:APC complex dissociates resulting in APC activation. Therefore, we introduced two alternative reaction pathways: In the first variant, we assume that the MCC:APC complex dissociate into MCC and APC (reaction Eq. (7^a^)), subsequently allowing the MCC to disassemble into its parts according to reaction Eq. (4) (Dissociation variant). In the second variant, the MCC component Cdc20 may stay in the complex with APC and only the further MCC complex members dissociate according to reaction Eq. (7^b^)(Convey variant).

**Figure 2:**
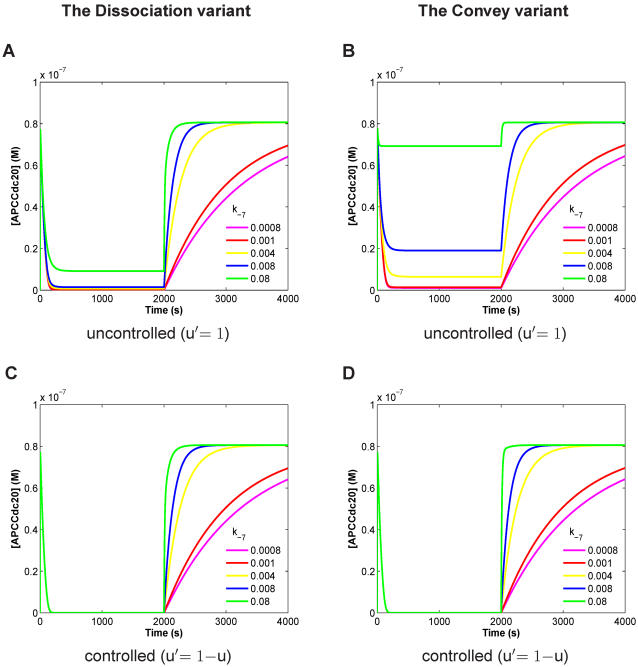
Dynamical behavior of APC:Cdc20 concentration versus time for the Dissociation variant (A, C) and the Convey variant (B, D) each in the uncontrolled (A, B) and the controlled (C, D) case. Calculation results are presented for different values of the rate k_−7_ in [s^−1^ >(dissociation of MCC:APC) between 0.0008 and 0.08, because k_−7_ is unknown and crucial for model behavior, as indicated. The APC:Cdc20 concentration should be close to zero before attachment and should rise quickly after attachment. Spindle attachment occurs at t = 2000s (switching parameter u from 1 to 0). For the uncontrolled case (A, B), both variants cannot explain the checkpoint behavior; and the Convey variant is even less satisfying compared to the Dissociation variant. In the controlled case (C, D), both variants fully inhibit APC:Cdc20 before attachment and both show fast switching recovery for high k_−7_ values. The controlled Convey variant (D) is slightly faster (by about 5 mins) in switching compared to the controlled Dissociation (C) variant. Parameters setting according to Table 1 and Table 2.

Figure 2 displays the APC:Cdc20 concentrations over time. For both, Dissociation and Convey variant, we have selected the time range such that each concentration can reach steady state. For all calculations, the concentrations and rates of Table 2 were chosen including those for k_7_, k_8_, and k_−8_. We varied the rate of k_−7_ (dissociation of MCC:APC) between 0.0008 and 0.08, because k_−7_ is unknown and crucial for model behavior. For both model variants, we distinguished 2 scenarios: in one scenario reaction Eq. (7^a^) (or Eq. (7^b^)) of the checkpoint is valid all the time (“uncontrolled”), while in the other case this reaction is silenced until it is activated by microtubule attachment to the kinetochore (“controlled”). This property of the controlled case is realized by introducing the factor u′ for reaction Eq. (7^a^) and Eq. (7^b^).

In the uncontrolled case, our model cannot explain the checkpoint behavior, independently of which pathway is chosen (Figure 2A–B). For the Dissociation variant, the APC:Cdc20 concentration is low for low values of k_−7_, however, in this case the switching recovery is unrealistically slow. On the other hand, for fast switching, k_−7_ must be high resulting in an increased APC:Cdc20 concentration before attachment (Figure 2A–B). This behavior is even worse for the Convey variant in the uncontrolled case (Figure 2B). For low values of k_−7_, both pathways behave rather similarly; for higher values of k_−7_ the Convey variant is even less satisfying compared to the Dissociation variant.

In the controlled case, we introduced the factor u′ (see above) regulating reaction Eq. (7^a^) and Eq. (7^b^), and re-calculated the model. Both pathways fully inhibit APC:Cdc20 before attachment and both show very fast switching recovery for high k_−7_ values (Figure 2C–D). Thus, a distinction between the two pathways in the controlled case is not possible based on our theoretical results. We observed that the controlled Convey variant is slightly faster (by about 5 mins) in switching compared to the controlled Dissociation variant. This makes the Convey variant slightly superior, however, we think that this difference is too small for a clear preference between the two pathways. Experimental measurements have to distinguish between these cases. Such experiments are in progress in our laboratory.

**Figure 3:**
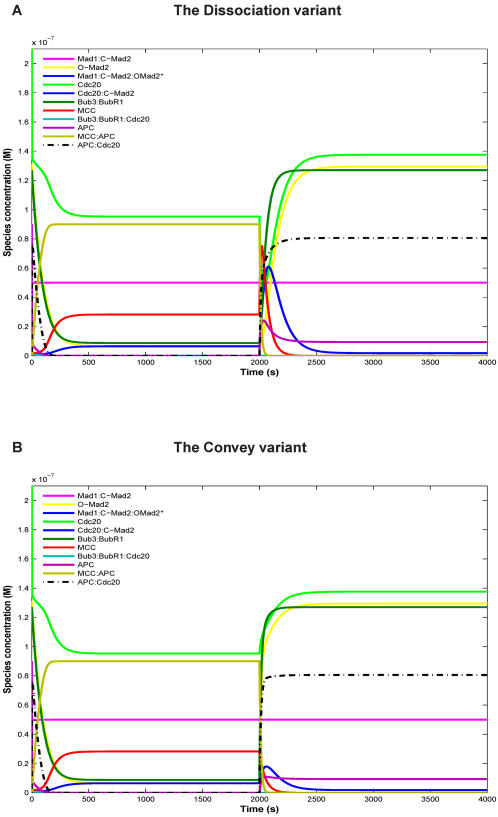
Species concentration over time for the controlled Dissociation variant (A) and the controlled Convey variant (B). Spindle attachment occurs at t = 2000s (switching parameter u from 1 to 0 and u' from 0 to 1). Both variants show similar qualitative dynamics. However, quantitative differences can be observed for species like Cdc20:C-Mad2 and MCC. Parameters setting according to Table 2 (“wild type”).

In addition to the APC:Cdc20 concentration values, we also analyzed the time-dependent concentrations of all reaction components. We observed differences between the two pathways in the controlled case for sub-complexes like Cdc20:C-Mad2 and MCC (Figure 3).

In our simulations, the MCC completely sequesters the APC so that no free APC is available until the microtubules are attached. Thus, Cdc20 has a dual function: until kinetochore attachment, Cdc20 contributes to MCC formation and thus APC inhibition, while after attachment Cdc20 acts as the APC activator.

### Model validation by mutation experiments

In order to validate our model, we tested different mutations (deletion and over-expression) of the proteins and complexes involved, measured in different organisms (Table 3).

**Table 3:** *In-silico* mutation experiments for validation

No.	Species	Organisms	Exp.	Experimental effects	Effects in our models
1.	Mad2	H. s.	D	-Impaired ^M^SAC [32]	- ^M^SAC fails to arrest
	Mad2	M.	D	-Unable to arrest [72]	& no Cdc20 sequestering.
	Mad2	H. s. & M.	D	-Defective ^M^SAC [73]	- [APC:Cdc20] very high.
	Mad2	H. s.	D	-Unable to bind Cdc20 or Mad1 [74]. More refs.: [31], [40], [108] [1], [34], [109]-[111].	
2.	Mad2	H.s.	O	-Activates the ^M^SAC [25]	-Activates the ^M^SAC
	Mad2	S.p.	O	-Blocks mitosis [90]	& full Cdc20 sequestering. -[APC:Cdc20] very low.
3.	Mad1	H.s.	D	-^ M^SAC inactivation & aneuploidy [75].	- ^M^SAC fails to arrest & no Cdc20 sequestering.
	Mad1	S.p.	D	- cell death [76]. More refs.: [77]-[79].	-[APC:Cdc20] very high.
4.	BubR1	H.s.	D	-Reduced ^M^SAC function, Reduced ^M^SAC binding to Cdc20:C-Mad2 [47].	-^M^SAC fails to arrest.
					-[APC:Cdc20] very high.
	BubR1	M.	D	-Increased polyploidy [82]. More refs.: [83], [84].	
5.	Bub3	M.	D	-Fails to arrest [80], [81].	-^M^SAC fails to arrest.
					-[APC:Cdc20] very high.
6.	Cdc20	S.c.	O	-Allows cells with a depolymerized spindle or damaged DNA to leave mitosis [91].	-^M^SAC fails to arrest.
					-[APC:Cdc20] very high.
				-Impairment MSAC and aneuploidization in oral cancer [112].	
7.	Cdc20	H.s.	D	-Reduced binding to Mad2, selective disruption from Mad2 [85].	- blocks mitosis.
					-[APC:Cdc20] very low.
	Cdc20	S.p.	D	-Arrest in metaphase [113].	
8.	Bub1	Drosophila	Inh.	-Chromosome missegregation [86].	-^M^SAC fails to arrest.
					-[APC:Cdc20] very high.
	Bub1	H.s.	Inh.	-Disruption of Bub3 localization, disruption of Bub3 binding to BubR1 [81].	
9.	Aurora B	Xenopus	Inh.	-Overriding the ^M^SAC function, perturbs MTs dynamics [87].	-^M^SAC fails to arrest.
					-[APC:Cdc20] very high.
	Aurora B	S.c.	Inh.	-Unregulated MTs,	
				-^ M^SAC fails to arrest [88], [89]	
10.	APC (units) Cdc26, apc9 Cdc6,doc1	S.p.	D	-Disruption of complex association [92], [93], More refs.: [114].	-Activates the^ M^SAC.
					-[APC:Cdc20] very low.

Abbreviations: D for deletion or knockdown experiments, O for over-expression experiments, and Inh. for inhibition; S.c., Saccharomyces cerevisiae; S.p., Schizosaccharomyces pombe; H. s., Homo sapiens(Human); and M., Murine.

Recent experimental studies report that deletion in different organisms of any ofMad2 [32], [74]–[76], Mad1 [77]–[81], Bub3 [82], [83], BubR1 [47], [84]–[86], Cdc20 [87], Bub1 [83], [88], or Aurora B [89]–[91] resulted in ^M^SAC defects like premature sister-chromatid separation, no mitotic arrest, reduced partner binding, increase of polyploidy, or death. Experimental details and our model predictions are in qualitative agreement as summarized in Table 3: For example, over-expression of Mad2 [25], [92] activates the ^M^SAC resulting in mitotic arrest, while over-expression of Cdc20 [93] allows cells to exit from mitosis, however with a depolymerized spindle or damaged DNA. Deletion of any of the APC subunits Cdc26, Apc9, Cdc6 or Doc1 disrupts complex association [94], [95] with no anaphase initiation. We observed that deletions and/or over-expression of proteins, realized experimentally or in our model, change checkpoint function in the same way.

**Figure 4:**
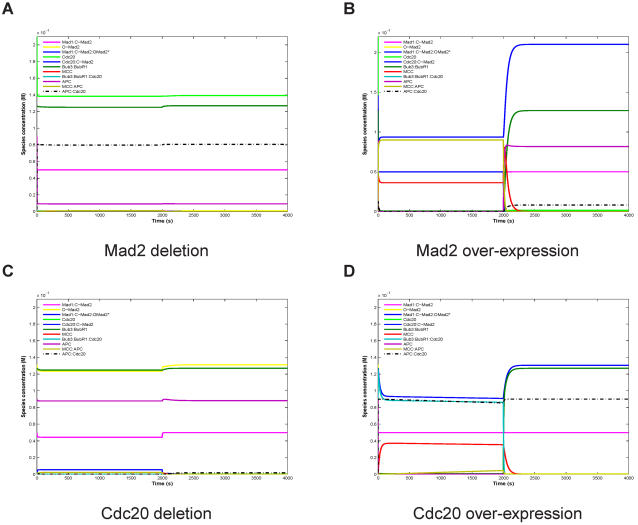
Simulation of Mad2 and Cdc20 mutations for the controlled Convey variant (cf. Table 3). For deletion we set the respective initial concentration 100 times lower, and for over-expression 10000 times higher. For proper functioning, APC:Cdc20 concentration should be very low(zero) before the attachment, and should increase quickly after attachment. Deletion of Mad2 (A) or Cdc20 (C) destroys the switching behavior, that is, the concentrations of all model species are rather constant. Mad2 deletion (A) causes high APC:Cdc20 concentration right from the beginning, while for Cdc20 deletion (C) APC:Cdc20 concentration is zero, by definition. For Mad2 over-expression (B) or Cdc20 over-expression (D), many species concentrations are affected. Particularly, for Mad2 over-expression (B) the APC:Cdc20 concentration remains low before attachment and, after attachment, stays significantly lower than in the wild type (meaning mitotic arrest). In contrast, for Cdc20 over-expression (D), the APC:Cdc20 concentration is high before attachment and also after attachment (meaning checkpoint failure). Spindle attachment occurs at t = 2000s (switching parameter u from 1 to 0 and u' from 0 to 1). Further setting as in Figure 2.

For the essential checkpoint proteins, Mad2 and Cdc20, we present the mutation effect on our model in detail in Figure 4 for the Convey variant. The effect is basically the same for the Dissociation variant (Figure S1). For Mad2 or Cdc20 deletion, the concentrations of all model components are rather stable, that is, they are almost not affected by microtubule attachment. However, in the case of Mad2 deletion, the APC:Cdc20 concentration is high (Figure 4A) while for Cdc20 deletion this concentration is zero by definition (Figure 4C). In the case of Mad2 or Cdc20 over-expression, many component concentrations were affected. In particular, for Mad2 over-expression the APC:Cdc20 concentration remains low before and significantly lower than in the wild type after attachment, explaining mitotic arrest and the delay of exit from mitosis (Figure 4B). In contrast, for Cdc20 over-expression, the APC:Cdc20 concentration is high before and after attachment (Figure 4D) resulting in total checkpoint failure. Thus, our ^M^SAC Model is able to explain the presented mutation phenotypes (Table 3, Figures 4, S1, S2, and S3).

## Discussion

Although our model is able to explain checkpoint function, this explanation does not contain details regarding the bio-molecular nature of the switching signal represented by the abstract factor u in our model. For a general explanation of mitosis it is desirable to replace the abstract factor “u” by chemical reactions of species like p31^comet^, Dynein, Usp44, and/or UbcH10. These species play a role in the signaling of the attachment to the ^M^SAC control network we modeled here. When further biochemical details become available, we will replace “u” by a network model encompassing these species. Other additional proteins and complexes are involved in ^M^SAC function implicitly. These species grant localization of outer kinetochore proteins as well as checkpoint proteins, which do not appear in our model explicitly. Examples are Bub1 (responsible for Bub3 and BubR1 localization [45], [96], [97]) and Mps1, an essential component of the ^M^SAC [43], [98]–[101] required for kinetochore localization of Mad1 and Mad2 [102]–[109]. Considering these additional proteins and their spatial localization would be an important next step towards a systems level model of mitosis.

## Supporting Information

Text S1 Supplement: Differential equations, Materials, Methods, and Optimization
(0.03 MB PDF)

Figure S1 Simulation of Mad2 and Cdc20 mutations for the controlled Convey Dissociation (cf. Table 3). For deletion we set the respective initial concentration 100 times lower, and for over-expression 10000 times higher. For proper functioning, APC:Cdc20 concentration should be very low(zero) before the attachment, and should increase quickly after attachment. Deletion of Mad2 (A) or Cdc20 (C) destroys the switching behavior, that is, the concentrations of all model species are rather constant. Mad2 deletion (A) causes high APC:Cdc20 concentration right from the beginning, while for Cdc20 deletion (C) APC:Cdc20 concentration is zero, by definition. For Mad2 over-expression (B) or Cdc20 over-expression (D), many species concentrations are affected. Particularly, for Mad2 over-expression (B) the APC:Cdc20 concentration remains low before attachment and, after attachment, stays significantly lower than in the wild type (meaning mitotic arrest). In contrast, for Cdc20 over-expression (D), the APC:Cdc20 concentration is high before attachment and also after attachment (meaning checkpoint failure). Spindle attachment occurs at t = 2000s (switching parameter u from 1 to 0 and u' from 0 to 1). Further setting as in Figure 2.
(0.69 MB TIF)

Figure S2 Simulation of BubR1, Aurora B, Mad1, and APC (subunits) mutations for the controlled Dissociation variant (cf. Table 3). For deletion we set the respective initial concentration 100 times lower, and for APC subunit 10 times lower. Spindle attachment occurs at t = 2000s (switching parameter u from 1 to 0 and u' from 0 to 1). Note that Bub3 deletion has the same effect like BubR1 (data not shown), and Bub1 deletion has the same effect like Aurora B (data not shown). APC:Cdc20 for the wild type should be very low (zero) before the attachment and increase quickly after attachment. Deletion of any of BubR1, Mad1, or Aurora B (as well as Bub3 and Bub1) results in high concentration of APC:Cdc20 right from the beginning (meaning checkpoint failure). Deletion of APC subunits disrupts the complex and thus makes APC:Cdc20 unavailable, which implies mitotic arrest. Parameter setting according to Table 2.
(0.74 MB TIF)

Figure S3 Simulation of BubR1, Aurora B, Mad1, and APC (subunits) mutations for the controlled Convey variant (cf. Table 3). The qualitative effect of the mutations is the same as for the Dissociation variant shown in Figure S2. There are quantitative differences in some species concentrations (c.f. Panal B, deletion of Aurora B). Same parameter settings as in Figure S2.
(0.72 MB TIF)
