# Supplementary material for: In-Silico Modeling of the Mitotic Spindle Assembly Checkpoint
Source: PLoS One. 2008 Feb 6;3(2):e1555. doi: 10.1371/journal.pone.0001555 (PMC2215771; doi:10.1371/journal.pone.0001555)
Supplement: Text S1 — Supplement: Differential equations, Materials, Methods, and Optimization (0.03 MB PDF) [file pone.0001555.s001.pdf]

B- ODEs of ^{M}SAC model Convey variant
