# Supplementary figures and images for: In-Silico Modeling of the Mitotic Spindle Assembly Checkpoint
Source: PLoS One. 2008 Feb 6;3(2):e1555. doi: 10.1371/journal.pone.0001555 (PMC2215771; doi:10.1371/journal.pone.0001555)

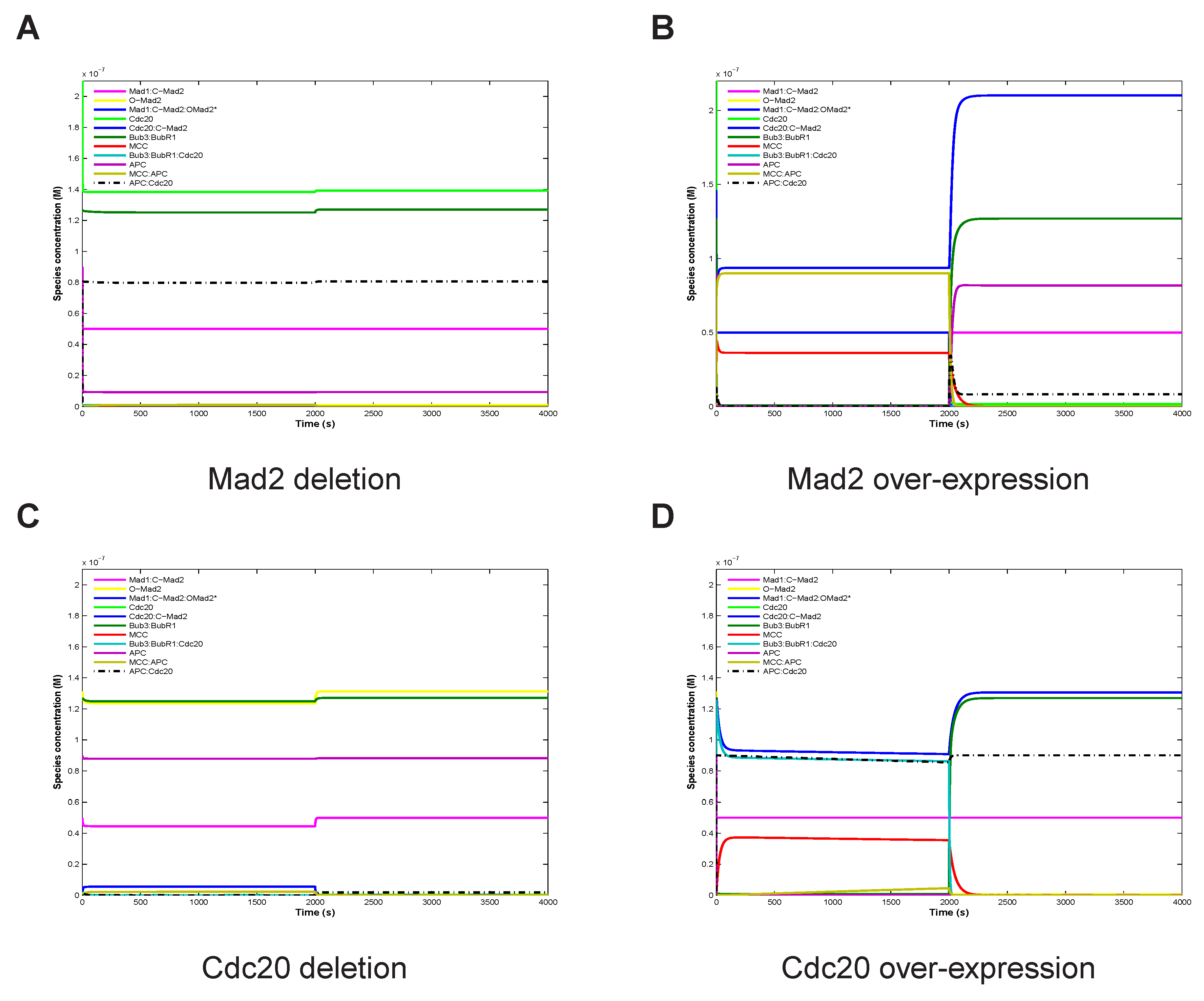

Supplement: Figure S1 — Simulation of Mad2 and Cdc20 mutations for the controlled Convey Dissociation (cf. Table 3). For deletion we set the respective initial concentration 100 times lower, and for over-expression 10000 times higher. For proper functioning, APC:Cdc20 concentration should be very low(zero) before the attachment, and should increase quickly after attachment. Deletion of Mad2 (A) or Cdc20 (C) destroys the switching behavior, that is, the concentrations of all model species are rather constant. Mad2 deletion (A) causes high APC:Cdc20 concentration right from the beginning, while for Cdc20 deletion (C) APC:Cdc20 concentration is zero, by definition. For Mad2 over-expression (B) or Cdc20 over-expression (D), many species concentrations are affected. Particularly, for Mad2 over-expression (B) the APC:Cdc20 concentration remains low before attachment and, after attachment, stays significantly lower than in the wild type (meaning mitotic arrest). In contrast, for Cdc20 over-expression (D), the APC:Cdc20 concentration is high before attachment and also after attachment (meaning checkpoint failure). Spindle attachment occurs at t = 2000s (switching parameter u from 1 to 0 and u' from 0 to 1). Further setting as in Figure 2. (0.69 MB TIF) [file pone.0001555.s002.tif]
